# Clinical, microbiological, and molecular characterization of pediatric invasive infections by *Streptococcus pyogenes* in Spain in a context of global outbreak

**DOI:** 10.1128/msphere.00729-23

**Published:** 2024-03-05

**Authors:** Eva Ramírez de Arellano, Jesús Saavedra-Lozano, Pilar Villalón, Ana Jové-Blanco, David Grandioso, Jared Sotelo, Anna Gamell, Juan José González-López, Eloísa Cervantes, María José Gónzalez, Victoria Rello-Saltor, Cristina Esteva, Francisco Sanz-Santaeufemia, Genoveva Yagüe, Ángela Manzanares, Patricia Brañas, Enrique Ruiz de Gopegui, Jaime Carrasco-Colom, Federico García, Emilia Cercenado, Isabel Mellado, Elena del Castillo, María Pérez-Vazquez, Jesús Oteo-Iglesias, Cristina Calvo, on behalf of the Spanish PedGAS-Net/CIBERINFEC GAS Study Group

**Affiliations:** 1 Laboratorio de Referencia e Investigación en Resistencia a Antibióticos e Infecciones Relacionadas con la Asistencia Sanitaria, Centro Nacional de Microbiología, Instituto de Salud Carlos III, Majadahonda, Madrid, Spain; 2 CIBER de Enfermedades Infecciosas (CIBERINFEC). Instituto Salud Carlos III, Madrid, Spain; 3 Servicio de Pediatría, Hospital General Universitario Gregorio Marañón. Universidad Complutense, Madrid, Spain; 4 Laboratorio de Referencia e Investigación en Taxonomía, Centro Nacional de Microbiología, Instituto de Salud Carlos III, Madrid, Spain; 5 Servicio de Microbiología, Hospital Universitario La Paz, Madrid, Spain; 6 Servicio de Enfermedades Infecciosas, Hospital San Joan de Déu, Barcelona, Spain; 7 Servicio de Microbiología, Hospital Universitario Vall d’Hebron, Barcelona, Spain; 8 Servicio de Pediatría, Hospital Virgen de la Arrixaca, Murcia, Spain; 9 Servicio de Microbiología, Hospital Universitario Niño Jesús, Madrid, Spain; 10 Servicio de Pediatría, Hospital Universitario Vall d’Hebron, Barcelona, Spain; 11 Servicio de Microbiología, Hospital San Joan de Dèu, Barcelona, Spain; 12 Servicio de Pediatría, Hospital Universitario Niño Jesús, Madrid, Spain; 13 Servicio de Microbiología, Hospital Virgen de la Arrixaca, Murcia, Spain; 14 Servicio de Pediatría, Hospital 12 de Octubre, Madrid, Spain; 15 Servicio de Microbiología, Hospital 12 de Octubre, Madrid, Spain; 16 Servicio de Microbiología, Hospital Universitario Son Espases, Instituto de Investigación Sanitaria Illes Balears (IdiSBA), Palma, Spain; 17 Servicio de Pediatría, Hospital Universitario Son Espases, Palma, Spain; 18 Servicio de Microbiología, Hospital San Cecilio, Instituto de Investigación IbS.GRANADA, Granada, Spain; 19 Servicio de Microbiología Clínica y Enfermedades Infecciosas, Hospital Universitario Gregorio Marañón, Madrid, Spain; 20 CIBER de Enfermedades Respiratorias (CIBERES). Instituto Salud Carlos III, Madrid, Spain; 21 Servicio de Pediatría y Enfermedades Infecciosas, Hospital Universitario La Paz, Fundación IdiPaz Madrid, Spain. Red de Investigación Traslación en Infectología Pediátrica (RITIP), Universidad Autónoma de Madrid, Madrid, Spain; 22 Servicio de Pediatría. Hospital Materno Infantil de Badajoz, Badajoz, Spain; Hackensack Meridian Health Center for Discovery and Innovation, Nutley, New Jersey, USA

**Keywords:** *Streptococcus pyogenes*, children, invasive disease, outbreak, GAS, M1_UK_

## Abstract

In December 2022, an alert was published in the UK and other European countries reporting an unusual increase in the incidence of *Streptococcus pyogenes* infections. Our aim was to describe the clinical, microbiological, and molecular characteristics of group A *Streptococcus* invasive infections (iGAS) in children prospectively recruited in Spain (September 2022–March 2023), and compare invasive strains with strains causing mild infections. One hundred thirty isolates of *S. pyogenes* causing infection (102 iGAS and 28 mild infections) were included in the microbiological study: *emm* typing, antimicrobial susceptibility testing, and sequencing for core genome multilocus sequence typing (cgMLST), resistome, and virulome analysis. Clinical data were available from 93 cases and 21 controls. Pneumonia was the most frequent clinical syndrome (41/93; 44.1%), followed by deep tissue abscesses (23/93; 24.7%), and osteoarticular infections (11/93; 11.8%). Forty-six of 93 cases (49.5%) required admission to the pediatric intensive care unit. iGAS isolates mainly belonged to *emm*1 and *emm*12; *emm*12 predominated in 2022 but was surpassed by *emm*1 in 2023. Spread of M1_UK_ sublineage (28/64 M1 isolates) was communicated for the first time in Spain, but it did not replace the still predominant sublineage M1_global_ (36/64). Furthermore, a difference in *emm* types compared with the mild cases was observed with predominance of *emm*1, but also important representativeness of *emm*12 and *emm*89 isolates. Pneumonia, the most frequent and severe iGAS diagnosed, was associated with the *spe*A gene, while the *ssa* superantigen was associated with milder cases. iGAS isolates were mainly susceptible to antimicrobials. cgMLST showed five major clusters: ST28-ST1357/*emm*1, ST36-ST425/*emm*12, ST242/*emm*12.37, ST39/*emm*4, and ST101-ST1295/*emm*89 isolates.

**IMPORTANCE:**

Group A *Streptococcus* (GAS) is a common bacterial pathogen in the pediatric population. In the last months of 2022, an unusual increase in GAS infections was detected in various countries. Certain strains were overrepresented, although the cause of this raise is not clear. In Spain, a significant increase in mild and severe cases was also observed; this study evaluates the clinical characteristics and the strains involved in both scenarios. Our study showed that the increase in incidence did not correlate with an increase in resistance or with an *emm* types shift. However, there seemed to be a rise in severity, partly related to a greater rate of pneumonia cases. These findings suggest a general increase in iGAS that highlights the need for surveillance. The introduction of whole genome sequencing in the diagnosis and surveillance of iGAS may improve the understanding of antibiotic resistance, virulence, and clones, facilitating its control and personalized treatment.

## INTRODUCTION

*Streptococcus pyogenes*, or group A *Streptococcus* (GAS), is one of the most frequent bacterial pathogens in the pediatric population, producing a wide array of mild and invasive diseases (1–3). *S. pyogenes* has a great pathogenic potential due to its capacity to carry multiple virulence factors (4, 5), including the surface M protein encoded by the *emm* gene. Several hypervirulent *S. pyogenes* lineages have emerged in high-income settings in the last decades, mainly the modern *emm*1 (M1_global_ clone) but also *emm*3, *emm*12, *emm*28, *emm*59, and *emm*89 (6). M1_global_ has been the major driver of invasive infections in Western countries since the mid-1980s, but reports in 2019 from the UK described the rapid emergence of a new *S. pyogenes emm*1 clonal lineage (M1_UK_) exhibiting enhanced expression of the superantigen SpeA (7, 8).

On 2 December 2022, an alert was published in the UK reporting an unusual increase in the incidence of *S. pyogenes* infections (mainly tonsillitis and scarlet fever) and, subsequently, invasive infections; no unusual *emm* types were detected (9, 10). Several countries in Europe rapidly reported a similar increase in streptococcal invasive infections (11–13). Pneumonia has probably been the clinical condition that increased the most in this epidemic outbreak (14). It is not clear if the incidence of *S. pyogenes* infections increased with a subsequent rise of the invasive forms, or if there was a replacement in the previously circulating strains with more virulent ones.

Our aim was to describe the clinical, microbiological, and molecular characteristics of GAS invasive infections (iGAS) in children prospectively recruited during the epidemic outbreak suffered in Spain between September 2022 and March 2023, and to compare invasive strains with a control group of strains causing mild infections.

## MATERIALS AND METHODS

### Study design, patients, and bacterial isolates

The European health alert secondary to the increase of pediatric iGAS motivated the Spanish Centro de Investigación Biomédica en Red for Infectious Diseases to promote a strategic research action to characterize these infections during this epidemic wave. The study was coordinated by the Spanish PedGAS-net, a multicenter network comprising 51 Spanish hospitals for the study of iGAS (Table S1) in patients ≤16 years.

Cases of iGAS were prospectively collected between September 2022 and March 2023. Clinical and epidemiological data were included in a RedCap (Research Electronic Data Capture) database. The *S. pyogenes* isolates were primarily obtained and identified by participating hospitals, and sent to the Centro Nacional de Microbiología (Surveillance Program for invasive infection by GAS) for typing, antibiotic susceptibility testing, and whole genome sequencing (WGS); only one isolate per patient was collected.

iGAS was defined according to the Centers for Disease Control and Prevention (CDC) (15) (Supplementary file 2). Epidemiology, clinical presentation, and outcome were evaluated.

Controls were collected in the same period at network centers from children with pharyngitis who met the Centor criteria (https://pap.es/articulo.php?lang=es&id=12162&term1=) and had a positive throat culture for *S. pyogenes*.

### Antimicrobial susceptibility and *emm* gene typing

Susceptibility to penicillin G, tetracycline, erythromycin, and clindamycin was performed using antibiotic gradient strips (Etest; bioMérieux, Durham, NC). The results were interpreted according to European Committee on Antimicrobial Susceptibility Testing (EUCAST) criteria (16). Macrolide resistance phenotypes were detected using the erythromycin-clindamycin double-disk test (17).

Typing of *emm* gene was performed using the protocols of the CDC (18).

### WGS of *S. pyogenes* isolates: genomic library preparation and sequence analysis

Genomic library preparation and sequence analysis were carried out as previously described (19). The quality of the short reads was assessed using FASTQC, and they were assembled into contigs with Unicycler 0.4.8 (20). The quality of the assembly was assessed with QUAST (http://quast.bioinf.spbau.ru/, accessed on June 2023). Prokka v1.14-beta (21) was used for automatic *de novo* assembly annotation. Raw sequence data were submitted to the European Nucleotide Archive (reference number: PRJEB67922).

### Phylogenetic analyses

Sequence types (STs) were calculated according to the multilocus sequence typing (MLST) scheme of the Public databases for molecular typing and microbial genome diversity (https://pubmlst.org/organisms/streptococcus-pyogenes) using Ariba v2.6.2 (22). Core genome MLST (cgMLST), consisting of 1,168 genes for *S. pyogenes* provided by SeqSphere+3.5.0 (Ridom, Münster, Germany), was performed. A simple diversity index (SDI) was applied to analyze population diversity (23).

Additionally, *emm*1 isolates from this study were analyzed as described by Linskey et al. (7) in comparison with a collection of 377 M1_global_ and 247 M1_UK_ hypervirulent *S. pyogenes* isolates reported in the previous reference. After removing all high single nucleotide polymorphisms (SNP) density regions (24), a core genome alignment with 6,411 SNPs was used to build a maximum-likelihood tree.

### Analysis of antimicrobial resistance and virulence genes

Antibiotic resistance genes were analyzed by Ariba v2.6.2 using the CARD database (https://card.mcmaster.ca, accessed on May 2023) and ResFinder [Center for Genomic Epidemiology (CGE) server, https://www.genomicepidemiology.org/services/, accessed on May 2023]. Virulence genes were analyzed with the previous methodology using the database Virulencefinder_db (https://bitbucket.org/genomicepidemiology/virulencefinder_db/src/master/, version 2022-12-02).

### Statistical analysis

Descriptive data were expressed as counts and percentages for categorical variables and as median, and first and third quartile [first, third Interquartile range (IQR)] for continuous variables. Categorical variables were compared using the chi-squared test or Fisher’s exact test, and results were expressed as odds ratio (OR). Continuous variables were analyzed with the Mann-Whitney U-test. Binary logistic regression modeling was used for multivariate analysis to determine risk factors associated with a worse clinical outcome. *P*-values <0.05 were considered statistically significant, and confidence intervals were calculated at 95% for all the estimations. Analyses were carried out using Statistical Package for the Social Sciences (SPSS) software (version 21; SPSS Inc., Chicago, IL, USA).

## RESULTS

### Participating hospitals and S. pyogenes isolates

A total of 130 isolates of *S. pyogenes* causing infection was included in the microbiological study; 102 were from cases (iGAS) and 28 were from controls. The 102 invasive isolates came from 20 hospitals from 10 Spanish autonomous communities (Table S1)

### Patients, clinical features, and risk factors

A total of 93 patients with iGAS and 21 controls with a mild disease accepted having their clinical data collected. More children were enrolled in 2023 (66; 71%). Median age was 43 months (IQR 15–74) for cases and 55 months (IQR 44–79) for controls; 43% of the cases (40/93) and 57% of the controls (12/21) were female, without significant differences.

Pneumonia was the most frequent clinical syndrome (41/93; 44.1%), followed by deep tissue abscesses (especially from ear, nose, and throat area and soft tissue) (23/93; 24.7%) and osteoarticular infections (11/93; 11.8%). Forty-six of 93 cases (49.5%) required admission to the pediatric intensive care unit (PICU) and 20 cases (21.5%) developed sepsis or septic shock during their evolution. In 36/93 (38.7%) cases, the infection developed bacteremia. Two children (2.2%) died of fulminant sepsis/toxic shock syndrome; one of them also died with pneumonia and pneumothorax.

The diagnosis of pneumonia was more frequent in 2022 (59.3% vs 36.9%; *P* = 0.049), with 9/41 (22%) cases developing bacteremia, but with 80.5% of them requiring PICU admission (OR = 11.1, CI = 4.08–30.21). In addition, 73.7% of patients who developed sepsis or septic shock required admission to the PICU (OR = 1.43, CI = 1.18–1.72). The diagnosis of deep tissue abscess was protective for PICU admission (OR = 0.678, CI = 0.52–0.88).

### Antimicrobial susceptibility and resistance genes by WGS

All 130 GAS isolates studied were susceptible to penicillin Minimum inhibitory concentration (MIC ≤0.032 mg/L). Global resistance to tetracycline, erythromycin, and clindamycin was 3.8% (5/130 isolates), 4.6% (6/130), and 3.8%, respectively. Resistance was higher in control isolates but without statistical significance (Table 1; Table S1).

**TABLE 1:** Main phenotypic and genotypic characteristics of *S. pyogenes* isolates from invasive infections and controls in this study

	Control isolates	Invasive isolates
Numbers of isolates	28	102
% Resistance		
Erythromycin	10.7	2.9
Tetracycline	7.1	2.9
Clindamycin	7.1	2.9
Numbers of *emm* types/subtypes	8	11
*emm* types more prevalent (*n*; %)	*emm*1 (*n* = 9; 32.1%); *emm*12 (6; 21.4%); *emm*89 (5; 17.9%)	*emm1* (55; 53.9%)*; emm12* (31; 30.4%)
*emm*1: M1_global_/M1_UK_	4/5	32/23
Number of STs	11	13
Average of isolates per ST (range)	2.5 (1–9)	7.8 (1–54)
SDI (range)	39.3	12.7
STs more prevalent (*n*; %)	ST28 (9; 32.1%); ST101 (4; 14.3%); ST36 (3; 10.7%).	ST28 (54; 52.9%); ST36 (23; 22.5%); ST242 (8; 7.8%)
Average number of virulence genes	5	5.7
Absence of hyaluronic acid capsule genes		
*has*A, *has*B, and *has*C (*n*; %)	10; 35.7%	8; 7.8%

All tetracycline-resistant isolates had the *tet*M gene. Macrolide resistance genes were *erm*B in three isolates showing the constitutive macrolide-lincosamide-streptogramin B (cMLSB) phenotype, *erm*T in two isolates with the inducible macrolide-lincosamide-streptogramin B (iMLSB) phenotype, and *mef* gene in one isolate with the M phenotype. *erm*B genes were from invasive isolates and *erm*T and *mef*A genes were from control isolates (Table S1).

Only tetracycline resistance was detected in three isolates (two *emm*22 and one *emm*60), and only erythromycin resistance was observed in four isolates (one *emm*1, two *emm*4, and one *emm*12 carrying *mef*A, *erm*T, and *erm*B, respectively). Tetracycline-erythromycin co-resistance was represented by *emm*12 and *emm*31 (one isolate each, both with *tet*M-*erm*B gene combination) (Table S1).

### *emm* gene typing and phylogenetic analysis by WGS

**Fig 1:**
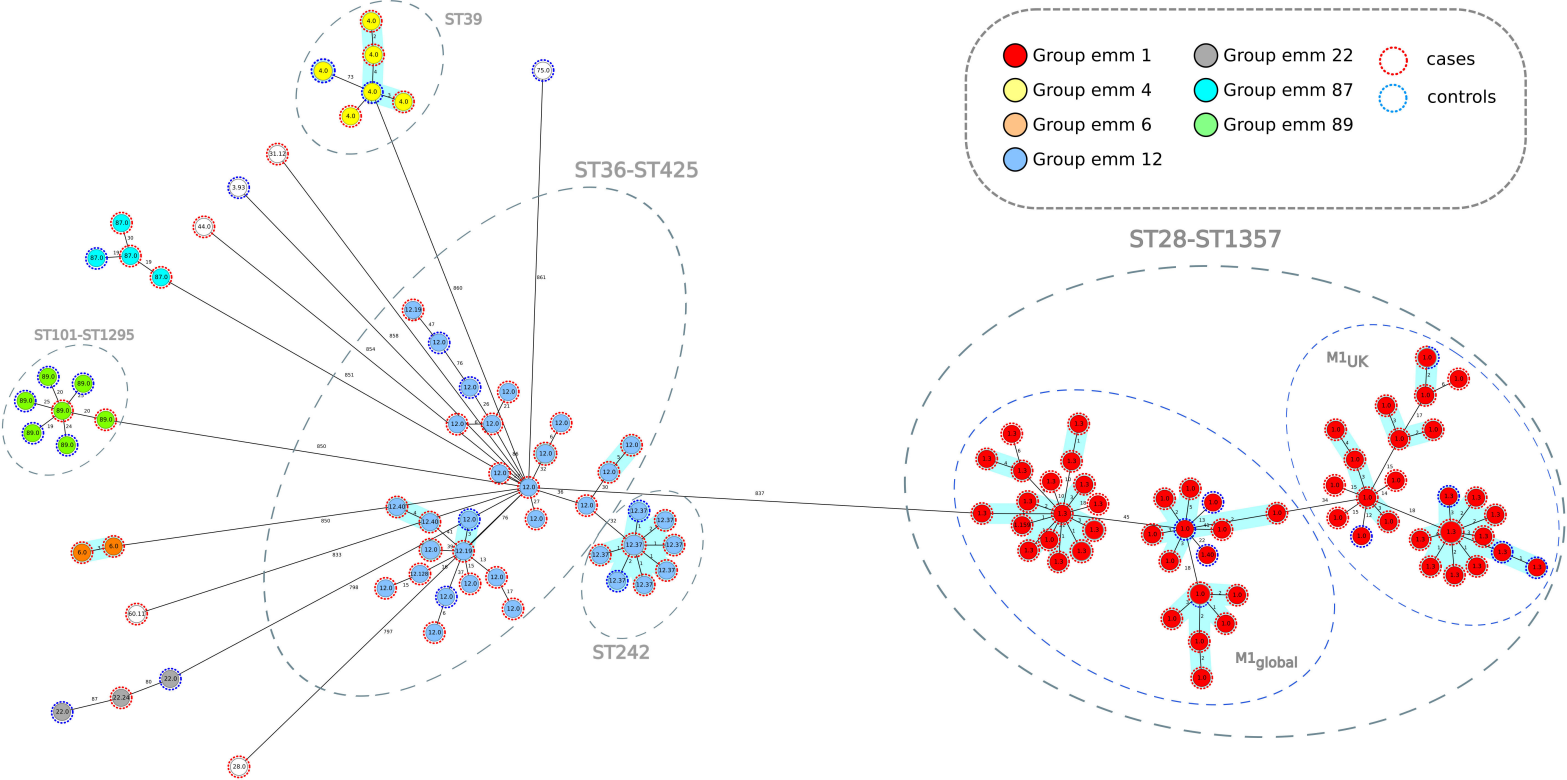
Population structure of *Streptococcus pyogenes* isolates from this study: minimum-spanning tree. Distances shown are based on cgMLST of 1168 genes using the parameter “pairwise ignoring missing values.” Fill colors in each circle indicate *emm* types, color of the dashed line in circles indicates the origin from cases or controls.

**Fig 2:**
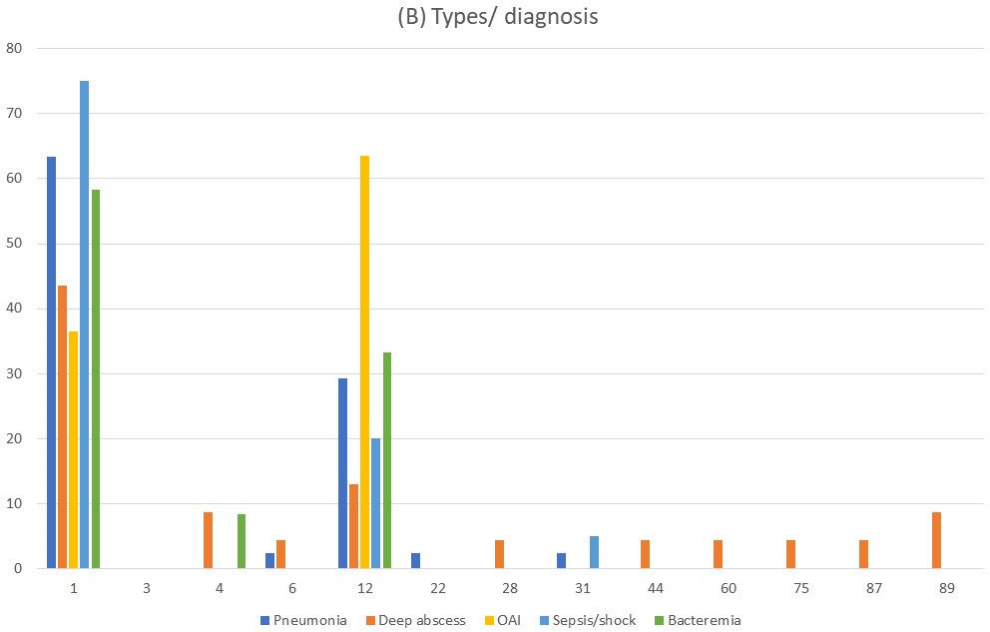
Types of *Streptococcus pyogenes* identified in patients with clinical data. (**A**) Types of controls compared to the total number of cases. (**B**) Types of cases by diagnoses.

**Fig 3:**
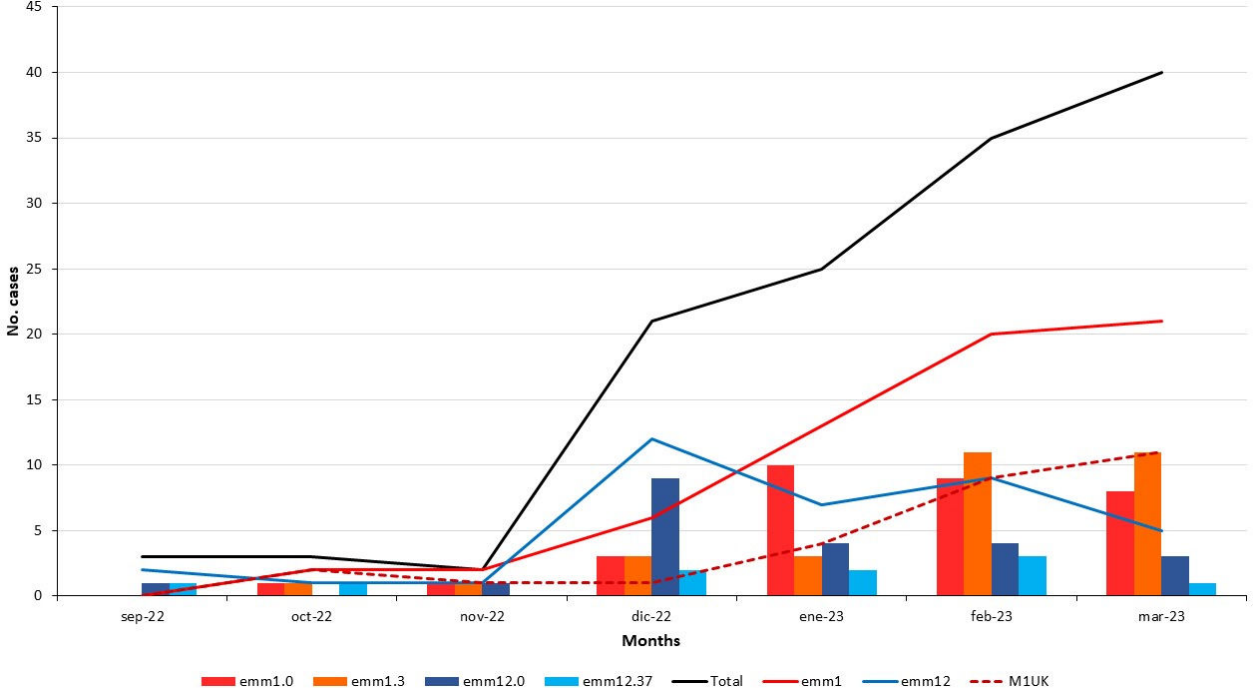
Evolution of the main clonal lineages of *Streptococcus pyogenes* during the 2022–2023 outbreak in Spain. Bar and linear chart that represents the temporal evolution of the total, *emm*1 (global plus UK clones), M1_UK_, *emm*1.0, *emm*1.3, *emm*12.0, and *emm*12.37 isolates. *y*-Axis represents the number of isolates and *x*-axis shows the studied period (months).

*emm* gene typing of 102 iGAS isolates revealed 11 *emm* types (Table S1), with *emm1* (53.9%) and *emm*12 (30.4%) predominating (Tables 1 and 2). Control isolates encompassed eight *emm* types, mainly *emm*1 (32.1%), *emm*12 (21.4%), and *emm*89 (17.9%). *emm*1 total isolates mostly included *emm*1.0 (50.0%) and *emm*1.3 (46.9%) subtypes, while *emm*12 isolates included *emm*12.0 (59.5%) and *emm*12.37 (27.0%) subtypes. The *emm*1.159, *emm*12.128, *emm*22.24, and *emm*31.12 subtypes (one isolate each) were first described in this study (Table 2; Fig. 1). Overall *emm* type diversity was compared between cases and controls showing significant differences (*P* = 0.006) (Fig. 2). Thus, *emm*1 and *emm*12 types were more frequent in cases, without statistical significance, but *emm*89 isolates were more common in the control group (17.9%) than in the invasive group (2%) (*P* = 0.005). Temporally, *emm*12 was more prevalent in late 2022, later surpassed by *emm*1 in the second part of the study period. Figure 3 shows the trend of the main clonal lineages of *emm*1 and *emm*12 during the outbreak.

**TABLE 2:** *emm* types/subtypes and the exotoxin gene profiles obtained in invasive and control *S. pyogenes* isolates

*emm* types/subtypes	STs correlation	*n* cases (%)/*n* controls (%)	Virulence profiles (*n*; %)
*emm1*		55 (53.9)/9 (32.1)	
*emm*1.0	ST28 (31), ST1357 (1)	27 (26.5)/5 (17.9)	*spe*A/*spe*G/*spe*J/*sme*Z (20/32; 62.5)
*emm*1.3	ST28	27 (26.5)/3 (10.7)	*spe*A/*spe*C/*spe*G/*spe*J/*sme*Z (26/30; 86.6)
*emm*1.159	ST28	1 (0.98)/0	*spe*A/*spe*C/*spe*G/*spe*J/*sme*Z (1/1; 100)
*emm*1.40	ST28	0/1 (3.6)	*spe*A/*spe*C/*spe*G/*spe*J/*sme*Z/*sic* (1/1; 100)
*emm*12		31 (30.4)/6 (21.4)	
*emm*12.0	ST36 (21), ST425 (1)	18 (17.6)/4 (14.3)	*spe*C/*spe*G/*speH*/*speI*/*sme*Z (15/22; 68.2)
*emm*12.19	ST36	2 (1.9)/0	*spe*G/*speH*/*speI*/*sme*Z (2/2; 100)
*emm*12.40	ST36	2 (1.9)/0	*spe*C/*spe*G/*speH*/*speI*/*sme*Z (2/2; 100)
*emm*12.37	ST242	8 (7.8)/2 (7.1)	*spe*C/*spe*G/*speH*/*speI*/*sme*Z (10/10; 100)
*emm*12.128	ST36	1 (0.98)/0	*spe*G/*speH*/*speI*/*sme*Z (1/1; 100)
*emm*89.0	ST101 (6), ST1295 (1)	2 (1.9)/5 (17.8)	*spe*C/*spe*G/ *sme*Z (6/7; 85.7)
*emm*87.0	ST62	3 (2.9)/1 (3.6)	*spe*C/*spe*G/*spe*J/*ssa*/*sme*Z (3/4; 75)
*emm*4.0	ST39	4 (3.9)/3 (10.7)	*spe*C/ *ssa*/*sme*Z (7/7; 100)
*emm*22			
*emm*22.0	ST46	0/2 (7.1)	*spe*C/*spe*G/*ssa*/*sme*Z (2/2; 100)
*emm*22.24	ST46	1 (0.98)/0	*spe*A/*spe*G/*ssa*/*sme*Z (1/1; 100)
*emm*6.0	ST382	2(1.9)/0	*spe*C/*spe*G/*speH*/*speI*/*sme*Z (2/2; 100)
Others: *emm*3.93, *emm*28.0, *emm*31.12, *emm*44.0, *emm*60.11, *emm*75.0	ST315, ST458, ST365, ST25, ST53, ST150	4 (3.9)/2 (7.1)	Presence of different and varied profiles (see supplementary material)

Using the MLST scheme, the 102 iGAS isolates grouped 13 STs with an SDI of 12.7 and an average of 7.8 isolates per ST (range = 1–54) (Table 1; Table S1). Control isolates were grouped into 11 STs with an SDI of 39.3 and an average of 2.5 isolates per ST (range = 1–9) (Table 1; Table S1). A strong correlation between STs and *emm* types was observed (Table 2; Fig. 1).

cgMLST analysis displayed a minimum-spanning tree (Fig. 1), identifying five major clusters with more than five isolates: ST28-ST1357/*emm*1 (*n* = 64), ST36-ST425/*emm*12 (*n* = 27), ST242/*emm*12.37 (*n* = 10), ST39/*emm*4 isolates (*n* = 7), and ST101-ST1295/*emm*89 (*n* = 7). Average allelic distances in pairwise comparisons of clustered isolates were 42 alleles (range: 0–71), 83 (range = 0–123), 2 (range = 0–4), 39 (range = 0–77), and 27 alleles (range = 19–34), respectively. These clusters included both iGAS and control isolates. ST1357, ST425, and ST1295 were single-locus variants of ST28, ST36, and ST 101, respectively, each represented by one isolate only.

**Fig 4:**
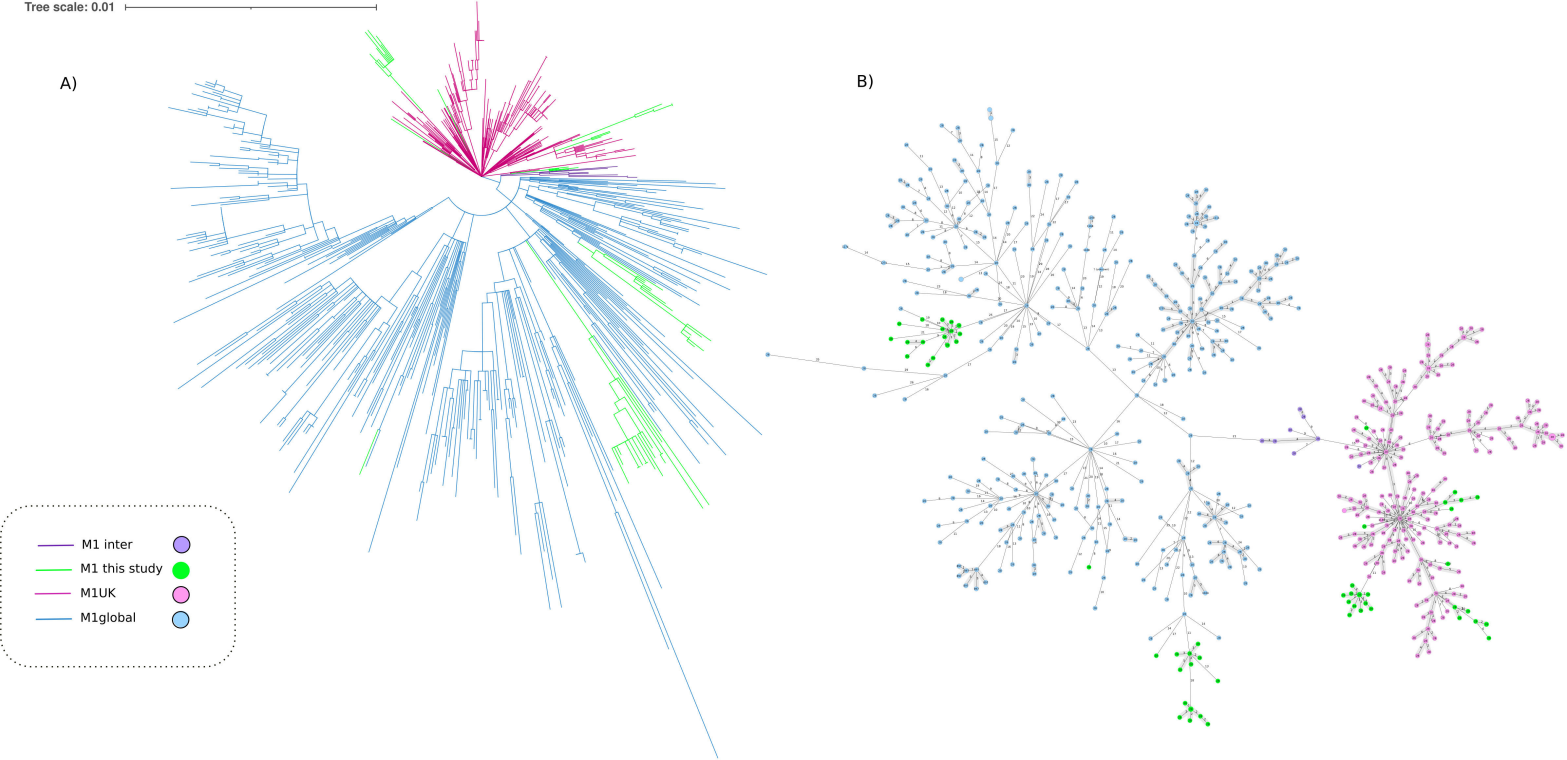
Population structure of *Streptococcus pyogenes* serotype M1 including isolates from this study and a well-characterized collection of 631 genomes that belong to M1_UK_, M1_global_, and M1inter (reference). (**A**) Maximum-likelihood tree showing the relationship between isolates; branch lengths are indicative of the number of SNPs. Colored branches indicate the variants of serotype M1. (**B**) Minimum-spanning tree showing distance based on cgMLST of 1,168 genes using the parameter “pairwise ignoring missing values.” Each circle is named with the MLST type of the isolates and colors indicate the variants of serotype M1.

Two international clonal lineages made up the *emm*1 population: M1_global_ (36 isolates, 56.3%) and M1_UK_ (28 isolates, 43.8%), both represented by *emm*1.0 and *emm*1.3 subtypes. Figure 4 presents joint cgMLST and SNP variability analysis of *emm*1 isolates from this study. The average SNP distance in pairwise comparison within the M1_UK_ group was 22 (range = 0–40). M1_UK_ isolates were found in both invasive (23/102) and control (5/28) groups, being isolated in children from six autonomous communities and 11 hospitals. The average SNP distance in pairwise comparison in Spanish strains grouped as M1_global_ was 34 (range: 0–66). Spanish isolates assigned as M1_UK_ differed from Spanish isolates grouped as M1_global_ by an average of 44 SNPs (range = 28–62).

### Superantigens and capsular genes in *S. pyogenes* identified by WGS

The exotoxin genes more frequently detected in the 102 iGAS isolates (presence in >50% of isolates) were streptococcal pyrogenic exotoxin genes *spe*G (97, 95.1%), *spe*C (70, 68.6%), *spe*J (62, 60.8%), and *spe*A (59, 57.8%); and the streptococcal mitogenic exotoxin gene *smeZ* (100, 98%) (Table S1). The streptococcal superantigen gene *ssa* was present in 12 (11.8%, five different *emm* types) of iGAS isolates.

*spe*A and *spe*I/*spe*H were highly associated with *emm*1 (55/59; 93.2%) and *emm*12 types (30/32; 93.7%), respectively. The virulent profiles belonging to the different STs/*emm* types are shown in Table 2. No differences in the virulence genes or in clinical severity were observed between M1_UK_ and M1_global_ isolates.

Eighteen isolates did not have the capsular genes *has*A, *has*B, and *has*C (Table 1); the absence of these genes was more frequent in control isolates (10/28; 35.7%) than in iGAS isolates (8/102; 7.8%) (*P* < 0.0006); *emm*4 and *emm*89 isolates were mainly implicated (7/18; 38.8%, for each one), but also *emm*22 (3/18, 16.7%) and *emm*31 (1/18, 5.6%).

### Joint analysis of clinical syndromes and microbiological features of pathogen strains

The most frequently identified *emm* types in children with iGAS cases were *emm*1 (49/93; 52.7%) and *emm*12 (29/93; 31.2%) (Fig. 2). Pneumonia was the most common clinical syndrome and it was caused almost exclusively by the types *emm*1 (26/41; 63.4%) and *emm*12 (12/41; 29.3%), with one case of *emm*6, *emm*22, and *emm*31, respectively. However, none of these types was significantly associated with pneumonia compared to other iGAS diagnoses. In deep tissue abscesses, practically all types were identified: 43.5% (10/23) were *emm*1, with only three cases of *emm*12. *emm*1 was the type more frequently associated with sepsis or septic shock (15/20, 75%; (OR = 1.27, CI = 1.03–1.58, *P* = 0.041).

No clinical or diagnostic differences were observed between isolates belonging to M1_UK_ and M1_global_.

Regarding the virulence genes, *speA* was significantly associated with the diagnosis of pneumonia (39/41 cases, 73.2%) (OR = 3.43, CI = 1.42–8.30). However, the *ssa* superantigen was only positive in one patient with pneumonia (2.5%) compared to nine (17.3%) without pneumonia (OR = 0.57 CI = 0.43–0.77). Thus, the presence of this superantigen made the development of pneumonia improbable. In addition, 66% (33/50) of children with the *spe*A gene required PICU admission, compared to 34% (17/43) who did not have it (OR = 2.14, CI = 1.27–3.59). Likewise, 62.0% (32/46) of patients admitted to the PICU had the *spe*J gene, compared to 38% (19/43) of the rest of the children (OR = 1.71, CI = 1.04–2.80, *P* = 0.34).

The virulence gene *spe*H was detected in 30 (32.3%) cases, compared with two (9.5%) controls (OR = 1.22, CI = 1.05–1.41). *spe*I was also associated with cases (OR = 1.22, CI = 1.05–1.41). However, the *ssa* superantigen was more frequent in controls (33%; 7/21) than in cases (10.8%; 10/93) (OR = 2.85, CI = 1.35–6.02).

In the multivariate analysis including all risk factors significantly associated with PICU admission (*spe*A, *spe*J, M1_UK_, necrotizing fasciitis/streptococcal toxic shock syndrome, pneumonia, and bacteremia), only pneumonia was an independent risk factor for that event (OR = 7.31, CI = 2.37–22.58).

## DISCUSSION

This study analyzes and characterizes iGAS in Spanish children during the epidemic peak detected in Europe at the end of 2022 and the beginning of 2023. A difference in serotypes was observed between mild cases and invasive infections, with a slightly higher prevalence of *emm*1 and *emm*12 in invasive infections than in mild cases, as well as a significant presence of *emm*89 in mild cases that was not detected in severe infections; in addition, the spread of M1_UK_ sublineage communicated for the first time in Spain. Pneumonia was the most frequent and severe invasive infection diagnosed, associated with the *spe*A virulence gene, while the *ssa* superantigen was associated with milder cases.

Although an increase in iGAS cases had already been described in the pre-pandemic years (25), this was clearly exceeded in the last aforementioned outbreak in both hemispheres (26–28). As in our series, the predominant clinical syndrome was complicated pneumonia (7, 9–26), which led to significant severity and the need for frequent admission to the PICU. Pneumonia was more common at the end of 2022, perhaps coinciding with the highest peak of respiratory viruses (13). Its incidence has been so high that, in some studies, *S. pyogenes* has surpassed *Streptococcus pneumoniae* as the first agent of pediatric bacterial pneumonia (29). Nevertheless, the invasive clinical syndromes typically produced by GAS were also observed in this study.

The most frequent types in children continued to be *emm*1 and *emm*12, both in our series and in that of other countries (26), as well as in many pre-pandemic pediatric cohorts (30). In our study, *emm*89 lacking the *has*ABC locus was one of the most frequent types observed in mild cases; this *emm* type, which belongs to clade 3 and emerged in 2008 (31), has been described as associated with dermal infections (4, 32).

In this study, *emm*12 was the second in frequency producing iGAS but with a clear temporal distribution, more frequent at the beginning of the study and being sequentially replaced by *emm*1 later on (Fig. 3). *emm*12.0/ST36 clone has been frequently detected in Spain (Surveillance Program for the invasive infection by group A Streptococcus, unpublished data Pilar Villalón); but the emergence of *emm*12.37/ST242 is noteworthy. Although we have not seen clinical differences between both *emm*12 clones, the evolution of *emm*12.37 must be monitored. Previously, *emm*12 was significantly associated with non-invasive infections in Denmark (27), although results in children with iGAS from Portugal during 2022/2023 were similar to the Spanish data (29).

In general, *S. pyogenes* isolated from controls was more diverse and different than that involved in iGAS, suggesting that some specific clones may have more capacity to produce invasive infections.

Invasive isolates were very susceptible to antibiotics, with a prevalence of resistance to erythromycin and clindamycin lower than those causing tonsillitis, although without significant differences. This rate of antibiotic resistance was also lower than the global figures recently published in Spain and Europe (4, 33, 34). A recent study with iGAS isolates from adults and children in Spain showed 11.8%, 8.9%, and 4.3% of tetracycline, erythromycin, and clindamycin resistance, respectively (4), while these figures were 40.8%, 20.4%, and 18.8% in Greek isolates (46.1% of them from children) from different types of infections (33). The spread of serotypes originally susceptible to antibiotics, such as the prevalent *emm*1 and *emm*12 in this outbreak, has previously been linked to the decrease of macrolides/lincosamides resistance in recent years (4, 35). Resistance to tetracycline was mainly represented by *emm*22, and resistance to erythromycin and clindamycin was represented by *emm*4 and *emm*12, which are three of the most common resistant *emm* types in Spain (34). *emm*11 and *emm*77 isolates were not detected; although these types are the main representative tetracycline and erythromycin co-resistant *emm* types in our country, they have mainly been associated with cutaneous infections in elderly people (4).

The spread of the new hypervirulent variant M1_UK_ of *S. pyogenes*, first described in the UK (7) and subsequently detected in other countries (8), was detected in this study for the first time in Spain. M1_UK_ has also demonstrated its high capacity for dissemination in Spain, although the replacement of M1_global_ by M1_UK_ has not yet been completed. In addition, contrary to what has been described in other studies (7, 8), greater clinical aggressiveness of M1_UK_ could not be demonstrated.

The strengths of this study include the enrollment of a large number of children with invasive infections at the height of the global GAS outbreak with high geographic representativeness, as well as the joint clinical and molecular bacterial analyses by complete genomic sequencing. A limitation of the study may be the small number of controls included.

In summary, our study shows that the increase in incidence does not seem to go with an increase in resistance or in a serotype shift. However, there seems to be a rise in severity, in part related to a greater rate of pneumonia diagnosis. The introduction of WGS in the diagnosis and surveillance of iGAS makes it possible to have precise molecular information on the genetic profile of antibiotic resistance, virulence, *emm* type, and clone that facilitates the implementation of personalized medicine against these infections. Continuous surveillance is required to promptly detect changes in epidemiological, clinical, and microbiological iGAS trends.

## Data Availability

Bacterial genome data (raw Illumina reads) are publicly available in the European Nucleotide Archive (ENA) (PRJEB67922).

## SUPPLEMENTAL MATERIAL

File S1 msphere.00729-23-s0001.docx PedGas Net working group.

File S2 msphere.00729-23-s0002.docx Clinical criteria.

Table S1 msphere.00729-23-s0003.xlsx Molecular, microbiological, and clinical data of the 130 *Streptococus pyogenes* isolates included in the study.

ASM does not own the copyrights to Supplemental Material that may be linked to, or accessed through, an article. The authors have granted ASM a non-exclusive, world-wide license to publish the Supplemental Material files. Please contact the corresponding author directly for reuse.
