# Supplementary material for: Clinical, microbiological, and molecular characterization of pediatric invasive infections by Streptococcus pyogenes in Spain in a context of global outbreak
Source: mSphere. 2024 Mar 5;9(3):e00729-23. doi: 10.1128/msphere.00729-23 (PMC10964401; doi:10.1128/msphere.00729-23)
Supplement: File S1 — PedGas Net working group. [file msphere.00729-23-s0001.docx]

**PEDGAS-NET working group:**

Cristina Calvo, Isabel Mellado, David Grandioso (Hospital La Paz, Madrid), Jesús Saavedra, David Aguilera, Elena Rincón, Ana Jové, Emilia Cercenado (Hospital General Universitario Gregorio Marañón; Madrid), Francisco José Sanz Santaeufemia, María José Gónzalez, Elena Sánchez (Hospital Niño Jesús, Madrid), Daniel Blázquez, Ángela Manzanares (Hospital 12 de Octubre, Madrid), Rut Del Valle (Hospital Infanta Sofía, San Sebastián de los Reyes), Elvira Cobo (Hospital Fundación de Alcorcón, Alcorcón), Gloria Caro (Hospital Infanta Elena, Valdemoro), Lucía Figueroa (Hospital General de Villalba, Villalba), Marta Llorente (Hospital Universitario del Sureste, Arganda), Pilar Galán (Hospital de Fuenlabrada, Fuenlabrada), Arantxa Berzosa, Marta Illán (Hospital Clínico San Carlos, Madrid), Cristina Comín, Katie Badillo (Hospital de Torrejón, Torrejón), Fátima Ara Montojo (Hospital Puerta de Hierro, Madrid), Ana Álvarez, Ignacio Callejas (Hospital de Getafe, Getafe), Natalia Cerdeira (Hospital Universitario del Henares, Coslada), Carmen Vázquez (Hospital Ramón y Cajal, Madrid), Jaime Carrasco (Hospital Son Espases, Palma de Mallorca), Manuel Oltra, Anabel Piqueras (Hospital La Fe, Valencia), Elena Montesinos (Consorcio Hospital General Universitario de Valencia, Valencia), Nuria Benavente (Hospital Clínico Universitario de Valencia, Valencia), César Gavilán (Hospital Universitario San Juan, Alicante), Marta Dapena (Hospital de Castellón, Castellón), Eloisa Cervantes, Ana Menasalvas, Genoveva Yagüe (Hospital La Arrixaca, Murcia), Concha Rex (Hospital Universitario Santa Lucía, Cartagena), Victoria Rello, Mayli Lung (Hospital Vall D´Hebron, Barcelona), Anna Gamell (Hospital San Joan De Déu, Barcelona), Nuria López (Hospital del Mar, Barcelona), Borja Guarch, Anna Hernández (Hospital Josep Trueta, Gerona), Berta Fernández, Carlos Rodrigo (Hospital Germans Trias i Pujol, Badalona), Olga Calavia (Hospital Joan XXIII, Tarragona), Lola Falcón (Hospital Virgen del Rocío, Sevilla), Leticia Martínez (Complejo Hospitalario Torrecárdenas, Almería), Laura Martín, Begoña Carazo (Complejo Hospitalario Regional de Málaga, Málaga), María Sánchez-Códez, Almudena Alonso (Hospital Universitario Puerta del Mar, Cádiz), Belén Sevilla, Federico García (Hospital San Cecilio, Granada), Beatriz Ruiz (Hospital Reina Sofía, Córdoba), Cristina Calvo Monge (Hospital Universitario Donostia, San Sebastián), María Itziar Pocheville (Hospital de Cruces, Bilbao), Laura Calle (Hospital Central de Asturias), Irene Rivero, Federico Martinón (Hospital Clínico Universitario, Santiago de Compostela) Elena Colino, Javier Cuenca (Complejo Hospitalario Materno-Insular, Las Palmas de Gran Canaria), Marta Pareja (Hospital General de Albacete, Albacete), Elena del Castillo (Hospital Materno Infantil de Badajoz, Badajoz), Beatriz Jiménez (Hospital Marqués de Valdecilla, Santander).
