# Supplementary material for: Clinical, microbiological, and molecular characterization of pediatric invasive infections by Streptococcus pyogenes in Spain in a context of global outbreak
Source: mSphere. 2024 Mar 5;9(3):e00729-23. doi: 10.1128/msphere.00729-23 (PMC10964401; doi:10.1128/msphere.00729-23)
Supplement: File S2 — Clinical criteria. [file msphere.00729-23-s0002.docx]

**Supplementary Appendix S1.** Definitions.

Invasive group A streptococcal disease: *Streptococcus pyogenes* isolated from a normally sterile site, such as blood, cerebrospinal fluid, pleural fluid, peritoneal fluid, pericardial fluid, bone, joint/synovial fluid, or internal body site (e.g., lymph node, brain) or from a wound culture accompanied by necrotizing fasciitis or *Streptococcal* toxic shock syndrome per Centers for Disease Control and Prevention.

Reference: Centers for Disease Control and Prevention. Active bacterial core surveillance: case definition and ascertainment. Available at: [https://www.cdc.gov/abcs/methodology/case-def-ascertain.html. Accessed 17 Januray 2023](https://www.cdc.gov/abcs/methodology/case-def-ascertain.html.%20Accessed%2017%20Januray%202023)).

Streptococcal Toxic Shock Syndrome (STSS): Clinical evidence of STSS included hypotension (less than the fifth percentile by age for children aged) and at least two of the following: renal impairment, coagulopathy, liver function abnormality, acute respiratory distress syndrome, generalized erythematous macular rash (that may desquamate) or soft-tissue necrosis (including necrotizing fasciitis, myositis or gangrene). Probable cases of STSS were defined as meeting clinical evidence in the absence of another identified etiology for the illness and with isolation of group A *Streptococcus* from a non-sterile site. Confirmed cases of STSS were defined as meeting clinical evidence with isolation of group A *Streptococcus* from a normally sterile site (e.g., blood or cerebrospinal fluid or, less commonly, joint, pleural, or pericardial fluid).

Reference: Centers for Disease Control and Prevention. Streptococcal Toxic Shock Syndrome (STSS) (*Streptococcus pyogenes*) 2010 Case Definition. Available at: <https://ndc.services.cdc.gov/case-definitions/streptococcal-toxic-shock-syndrome-2010/>.
